# Incidence, clinical characteristics, and outcome after unexpected cardiac arrest among critically ill adults with COVID-19: insight from the multicenter prospective ACICOVID-19 registry

**DOI:** 10.1186/s13613-021-00945-y

**Published:** 2021-11-13

**Authors:** Jonathan Chelly, Gaetan Plantefève, Toufik Kamel, Cédric Bruel, Saad Nseir, Christopher Lai, Giulia Cirillo, Elena Skripkina, Sébastien Ehrminger, Fernando-Daniel Berdaguer-Ferrari, Julien Le Marec, Marine Paul, Aurélie Autret, Nicolas Deye, the ACICOVID-19 study group, Jean-Michel Arnal, Julio Badie, Audrey Berric, Jennifer Brunet, Thibault Bertrand, Dorothée Carpentier, Karim Chaoui, Anaïs Chapelle, Riad Chelha, Gaëlle Corno, Cédric Daubin, Richard Descamps, Alexandre Demoule, Stéphanie Deryckere, Stephane-Yannis Donati, Laurent Ducros, Nathalie Embriaco, Nicolas Engrand, Camille Foucault, Sean Anthony Freeman, Santiago Freita Ramos, Arnaud Galbois, Aude Garnero, Cyrille Geay, Laurent Guérin, Vivien Hong Tuan Ha, Thomas Hullin, Sébastien Jochmans, Michel Kaidomar, Charlotte Kelway, Marie Labruyere, Romaric Larcher, Stéphane Legriel, Maxime Leloup, Olivier Lesieur, Isabelle Malissin, Sandie Mazerand, Bruno Mégarbane, Marie-Anne Mélone, Edouard Menoret, Matthieu Metzelard, Nicolas Mongardon, Ly Van Phack Vong, Romain Persichini, Nicolas Pichon, Santiago Picos Gil, Jean-Pierre Quenot, Damien Roux, David Schnell, Florian Sigaud, Clement Suply, Benjamin Sztrymf, Nicolas Terzi, Didier Thevenin, Sebastian Voicu

**Affiliations:** 1 Intensive Care Unit, Centre Hospitalier Intercommunal Toulon-La Seyne sur Mer, Hôpital Sainte Musse, 54 rue Henri Sainte Claire Deville, 83056 Toulon, France; 2 Intensive Care Unit, Centre Hospitalier Victor Dupouy, Argenteuil, France; 3 Intensive Care Unit, Centre Hospitalier Régional d’Orléans, Orléans. Inserm UMR1153, ECSTRRA, Université de Paris, Paris, France; 4 Intensive Care Unit, Groupe Hospitalier Paris Saint Joseph, Paris, France; 5 Médecine Intensive Réanimation, CHU Lille, Inserm U1285, Université de Lille, CNRS, UMR 8576-UGSF, Unité de Glycobiologie Structurale et Fonctionnelle, Lille, France; 6 Medical Intensive Care Unit, Hôpital de Bicêtre, Université Paris-Saclay, Assistance Publique Hôpitaux de Paris (AP-HP), Le Kremlin-Bicêtre, France; 7 Intensive Care Department, Groupe Hospitalier Sud Ile de France, Melun, France; 8 Service d’anesthésie-réanimation chirurgicale, DMU CARE, DHU A-TVB, Assistance Publique Hôpitaux de Paris (AP-HP), Hôpitaux Universitaires Henri Mondor, Créteil, France; 9 Intensive Care Unit, Grand Hôpital de l’Est Francilien-site de Marne la Vallée, Jossigny, France; 10 Intensive Care Unit, Hôpital Nord Franche-Comté, Trévenans, France; 11 Intensive Care Unit, Département R3S, Pitié-Salpétrière hospital, Assistance Publique Hôpitaux de Paris (AP-HP), Sorbonne Université, Paris, France; 12 Intensive Care Unit, Centre Hospitalier de Versailles-site André Mignot, Le Chesnay, France; 13 Clinical Research Department, Centre Hospitalier Intercommunal Toulon-La Seyne sur Mer, Toulon, France; 14 Medical and Toxicological Intensive Care Unit, Inserm U942, Assistance Publique Hôpitaux de Paris (AP-HP), Centre Hospitalier Universitaire Lariboisière, Paris, France

**Keywords:** In-hospital cardiac arrest, Intensive care unit, SARS-CoV-2, COVID-19

## Abstract

**Background:**

Initial reports have described the poor outcome of unexpected cardiac arrest (CA) in intensive care unit (ICU) among COVID-19 patients in China and the USA. However, there are scarce data on characteristics and outcomes of such CA patients in Europe.

**Methods:**

Prospective registry in 35 French ICUs, including all in-ICU CA in COVID-19 adult patients with cardiopulmonary resuscitation (CPR) attempt. Favorable outcome was defined as modified Rankin scale ranging from 0 to 3 at day 90 after CA.

**Results:**

Among the 2425 COVID-19 patients admitted to ICU from March to June 2020, 186 (8%) experienced in-ICU CA, of whom 146/186 (78%) received CPR. Among these 146 patients, 117 (80%) had sustained return of spontaneous circulation, 102 (70%) died in the ICU, including 48 dying within the first day after CA occurrence and 21 after withdrawal of life-sustaining therapy. Most of CA were non-shockable rhythm (90%). At CA occurrence, 132 patients (90%) were mechanically ventilated, 83 (57%) received vasopressors and 75 (51%) had almost three organ failures. Thirty patients (21%) had a favorable outcome. Sepsis-related organ failure assessment score > 9 before CA occurrence was the single parameter constantly associated with unfavorable outcome in multivariate analysis.

**Conclusions:**

In-ICU CA incidence remains high among a large multicenter cohort of French critically ill adults with COVID-19. However, 21% of patients with CPR attempt remained alive at 3 months with good functional status. This contrasts with other recent reports showing poor outcome in such patients.

*Trial registration*: This study was retrospectively registered in ClinicalTrials.gov (NTC04373759) in April 2020 (https://www.clinicaltrials.gov/ct2/show/NCT04373759?term=acicovid&draw=2&rank=1).

**Supplementary Information:**

The online version contains supplementary material available at 10.1186/s13613-021-00945-y.

## Introduction

Before the Coronavirus disease 2019 (COVID-19) outbreak, incidence and mortality of unexpected cardiac arrest (CA) occurring in intensive care unit (ICU) varied widely according to clinical setting [1–4]. Since the beginning of the COVID-19 outbreak, small or monocentric reports from China and the USA, have uniformly described the dramatically poor outcome of patients hospitalized for COVID-19 pneumonia (including patients admitted to general/unmonitored wards or to ICU) who experienced in-hospital cardiac arrest (IHCA) [5–8]. Among these reports, IHCA incidence was around 20% [5] and survival with good neurological status ranged from 0 to 0.7%, leading to discussions regarding the futility of cardiopulmonary resuscitation (CPR) attempt in such patients [9–15]. More recently, the authors of a large multicenter study have reported a 14% incidence of in-ICU CA among 5019 patients admitted for severe COVID-19 in the USA. Among the 400 patients with CPR attempt included in this cohort, survival to hospital discharge and survival with good neurological status were only 12 and 7%, respectively [16]. In another North American multicenter cohort of 260 IHCA patients, survival at hospital discharge reached 15% among the 166 patients who experienced in-ICU CA [17]. These outcomes warrant further investigations into the risks and benefits of performing CPR in such patients, especially because the CPR process could generate an important risk of transmission of COVID-19 for healthcare professionals [18]. Moreover, there are currently scarce data regarding the characteristics and outcomes of in-ICU CA in patients with COVID-19 in Europe [19].

This French multicenter cohort study was designed to report the incidence of in-ICU CA among adults with confirmed COVID-19 pneumonia, and to report the outcome of patients experiencing such CA with CPR attempt.

## Methods

### Study setting and population

Thirty-five French ICUs participated to the ACICOVID-19 registry (see Additional file 1: Table S1 in the online supplemental data describing ICUs characteristics). The ethical board of the French Intensive Care Society approved the study that was registered in ClinicalTrials.gov (NTC04373759). The number of patients admitted in ICU with a laboratory-confirmed COVID-19 pneumonia from March 1st to June 30th, 2020 (i.e., the first French wave), was prospectively reported by each center, while all patients experiencing in-ICU CA were included. In-ICU CA was defined as patients experiencing an unexpected CA occurring during the ICU stay and requiring CPR attempt, regardless of sustained return of spontaneous circulation (ROSC) achievement. Patients who did not receive CPR (because of a “do-not-resuscitate” order, a withdrawal of life-sustaining therapy [WLST] decision, or CPR considered as futile when death was expected because of end-stage or multi-organ failure) were excluded. All patients or relatives were informed and approved the inclusion of their data in the ACICOVID-19 registry.

### Data collection

All data on CA were prospectively recorded as previously recommended following the Utstein guidelines [20]. If a patient experienced more than one CA, only the first was considered. CA characteristics were recorded as follows: last available clinical and biological parameters, sepsis-related organ failure assessment score (SOFA) and organ failures (defined as SOFA organ sub-score ≥ 3) within the hour before CA occurrence, ongoing treatments at the time of CA, time from collapse to CPR (no-flow duration corresponding to the measured—using the monitor—or the estimated duration between the ventricular fibrillation/tachycardia or asystole alarm and the first CPR initiation) and from first CPR to ROSC (low-flow duration), CPR management, and suspected or confirmed etiology of the in-ICU CA. Sustained ROSC was defined as the possibility to maintain ROSC with an identifiable pulse for more than 20 min [20].

### Objectives and outcome variables

We aimed to report the incidence of in-ICU CA among patients admitted for COVID-19 pneumonia, and the clinical characteristics and outcome of such CA patients. The incidence of in-ICU CA was defined as the ratio between the number of in-ICU CA occurrence and the number of patients admitted for COVID-19 in participating ICUs. Vital and functional status were collected using the modified Rankin scale (mRS) [21] at ICU discharge and at day 90 after CA. Favorable and unfavorable outcomes were defined as mRS ranging from 0 to 3 and from 4 to 6, respectively. Our secondary objective was to identify risk factors associated with an unfavorable outcome at day 90 after in-ICU CA. Additionally, to better define patients’ long-term outcome, the mRS at day 180 after CA was also collected.

### Statistical analysis

Categorical variables were expressed as number and proportions (%) and compared using either a Chi-square test or a Fisher’s exact test. Continuous variables were expressed as median [25–75% interquartile range] and compared using Student’s *t* test or nonparametric Mann–Whitney test if needed. A multivariate logistic regression was performed to investigate factors associated with unfavorable outcome at day 90 after CA. According to our limited cohort size, factors of clinical relevance were selected among all significant univariate factors with *p* ≤ 0.01 and included in the initial model. A stepwise backward elimination approach that gradually reduces the initial model was used. Variables that did not achieve statistical significance in multivariate analysis were removed from the model. Odds ratios (OR) were expressed with their 95% confidence intervals (CI). Significance of all statistical tests was considered with *p* < 0.05. All analyses were performed using SAS^®^ version 9.4 (SAS Institute, Cary, NC, USA).

## Results

### In-ICU CA incidence

**Fig. 1:**
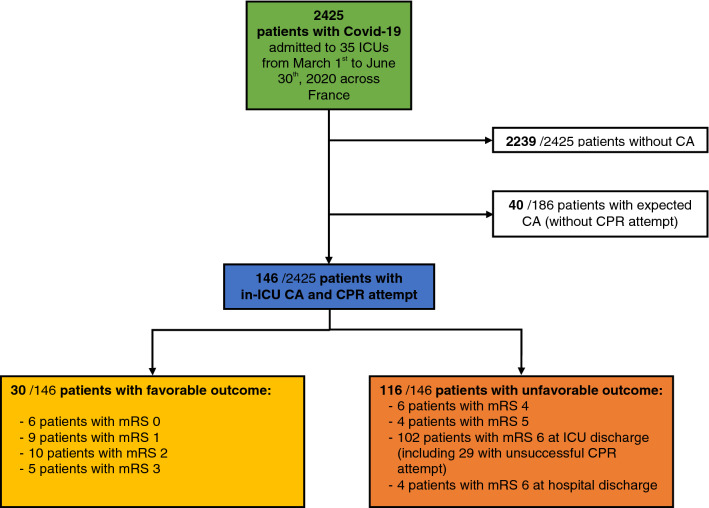
Study flowchart.* ICU* intensive care unit,* CA* cardiac arrest,* mRS* modified Rankin scale

Among the 2425 patients admitted to the 35 participating ICUs with confirmed COVID-19 pneumonia during the study period, 186 (8%) experienced CA (including 18 patients with CA occurring the first day after ICU admission), 146 (6%) patients had CPR attempted and 117 (5%) patients had sustained ROSC (Fig. 1). CA incidence varied from 2 to 20% of patients admitted with COVID-19 in the 35 participating ICUs, with a median incidence of 6% [4–10]. As detailed in the Additional file 1: Figure S1 (online supplemental data), the younger was the patient the most frequent was the CPR attempt.

### Patients’ characteristics at ICU admission

General characteristics at ICU admission of the 146 in-ICU CA patients with CPR attempt according to outcome are provided in Table 1 and Additional file 1: Table S2 in the online supplemental data. Median age was 62 years [55–70], BMI 29 kg/m^2^ [26–33], and the female/male ratio was 1/5. At ICU admission, median simplified acute physiology score 2 and SOFA were 42 [32–57] and 7 [4–11], respectively. Respiratory, neurologic, and circulatory failure were the most frequent organ failures at ICU admission.

**Table 1:** Main general characteristics at ICU admission of overall patients and according to outcome at day 90 after in-ICU CA

Characteristic	All patients (*N* = 146)	Favorable (*N* = 30)	Unfavorable (N = 116)	*p *value
Demographics				
Age, years	62 [55–70]	61 [54–69]	63 [56–69]	0.51
Male gender	118 (81)	25 (83)	93 (80)	0.70
Body mass index, kg/m^2^	29 [26–33]	30 [26–33]	29 [26–33]	0.66
Previous comorbidities				
Hypertension	80 (55)	12 (40)	78 (67)	0.07
Diabetes	49 (34)	5 (17)	44 (38)	0.03
Respiratory disease	17 (12)	4 (13)	13 (11)	0.75
Malignancy	15 (10)	2 (7)	13 (11)	0.47
Ischemic heart disease	13 (9)	2 (7)	11 (9)	0.63
Moderate to severe chronic kidney disease^a^	8 (5)	2 (7)	6 (5)	0.75
Chronic heart failure	2 (1)	1 (3)	1 (1)	0.30
Charlson comorbidity index	3 [2–4]	2 [1–4]	3 [2–4]	0.20
Chronic previous treatments				
Angiotensin conversion enzyme/receptor blocker	51 (35)	7 (23)	44 (38)	0.14
Calcium channel blocker	31 (21)	2 (7)	29 (25)	0.03
Beta blocker	23 (16)	0 (0)	23 (20)	0.008
Corticosteroid	8 (5)	0 (0)	8 (7)	0.14
Severity of illness at ICU admission				
SAPS-2	42 [32–57]	41 [32–44]	43 [30–61]	0.14
SOFA	7 [4–11]	6 [4–10]	7 [4–12]	0.41
Organ failures (SOFA organ sub-score ≥ 3)				
Respiratory	104 (71)	21 (70)	83 (72)	0.30
Neurologic	47 (32)	10 (33)	37 (32)	0.82
Circulatory	47 (32)	8 (27)	39 (34)	0.27
Renal	22 (15)	3 (10)	19 (16)	0.28
Hematologic	2 (2)	0 (0)	2 (2)	0.44
Hepatic	1 (1)	0 (0)	1 (1)	0.59
SOFA > 9	30 (21)	8 (27)	22 (19)	0.34

Results are expressed as *N* (%) or median [25–75% interquartile range] unless expressed otherwise. Favorable outcome: modified Rankin scale ranging from 0 to 3 at day 90 after CA; unfavorable outcome: modified Rankin scale ranging from 4 to 6 at day 90 after CA; ICU: intensive care unit; CA: cardiac arrest; SAPS-2: simplified acute physiology score 2; SOFA: sepsis-related organ failure assessment score

^a^Moderate = creatinine > 3 mg/dl (270 μmol/l); severe = on dialysis

Patients with an unfavorable outcome had more diabetes and more previous chronic treatment with calcium channel blockers and betablockers than those with favorable outcome (25 vs. 7%, and 20 vs. 0%, respectively).

### In-ICU CA history and characteristics

Patients’ characteristics at in-ICU CA occurrence according to outcome are described in Table 2 and Additional file 1: Table S3 in the online supplemental data. At the time of in-ICU CA, SOFA was 11 [7–15] and 75 patients (51%) suffered from almost three organ failures. The median delays between in-ICU cardiac arrest occurrence and the first COVID-19 symptoms, the hospital admission, and the ICU admission were 21 days [14–31], 13 days [6–24], and 11 days [5–22], respectively. In-ICU CA mostly occurred after non-shockable rhythm (90%), with witnessed CA (89%), and immediate CPR (median no-flow interval 0 min [0–0]) with a low-flow duration of 4 min [2–10].

**Table 2:** Main patients’ characteristics at in-ICU cardiac arrest occurrence according to outcome at day 90

Characteristic	All patients (*N* = 146)	Favorable (*N* = 30)	Unfavorable (*N* = 116)	*p *value
Severity of illness at the time of CA				
SOFA score	11 [7–15]	7 [4–9]	12 [8–15]	< 0.001
Organ failures (SOFA organ sub-score ≥ 3)				
Respiratory	98 (67)	16 (53)	82 (71)	0.001
Neurologic	91 (62)	15 (50)	76 (66)	0.007
Circulatory	64 (44)	8 (27)	66 (57)	0.003
Renal	47 (32)	4 (13)	43 (37)	0.002
Hepatic	4 (3)	0 (0)	4 (3)	0.28
Hematologic	2 (1)	0 (0)	2 (2)	0.46
SOFA > 9	81 (55)	7 (23)	74 (64)	< 0.001
CA history				
Interval from Covid-19 first symptoms to CA, days	21 [14–31]	25 [17–37]	20 [13–31]	0.04
Interval from hospital admission to CA, days	13 [6–24]	19 [10–30]	12 [6–23]	0.04
Interval from ICU admission to CA, days	11 [5–22]	15 [7–29]	9 [5–21]	0.07
Witnessed CA	130 (89)	28 (93)	102 (88)	0.66
Immediate bystander CPR	125 (86)	28 (93)	97 (83)	0.28
VT/VF	14 (10)	5 (17)	9 (8)	0.14
No-flow duration, min	0 [0 – 0]	0 [0 – 0]	0 [0–0]	0.53
Low-flow duration, min	4 [2–10]	3 [1–5]	5 [2–13]	0.004
Total epinephrine bolus dose, mg	2 [1–5]	1 [1–2]	2 [2–6]	< 0.001
Intubation during or immediately after CPR	22 (15)	7 (23)	15 (13)	0.17
CA circumstances				
At night or during weekend	101 (69)	24 (80)	77 (66)	0.15
≤ 24 h after ICU admission	17 (12)	2 (7)	15 (13)	0.34
Nursing procedure	11 (8)	4 (13)	7 (6)	0.18
Immediately after ICU admission	6 (4)	0 (0)	6 (5)	0.20
During intra-hospital transfer	2 (1)	0 (0)	2 (2)	0.47
Ongoing treatments at the time of CA				
Mechanical ventilation	132 (90)	25 (83)	97 (84)	0.68
Vasopressors	83 (57)	12 (40)	61 (53)	0.04
Renal replacement therapy	27 (18)	1 (3)	26 (22)	0.02
Prone position	24 (16)	2 (7)	22 (19)	0.11
ECMO	13 (9)^a^	2 (7)	11 (9)	0.63
Last available parameters at the time of CA^b^				
Mean arterial pressure, mmHg	76 [63–91]	86 [77–94]	73 [60–87]	< 0.001
Heart rate, bpm	94 [79–110]	92 [82–107]	95 [78–110]	0.84
FiO_2_, %	71 [40–100]	40 [31–60]	90 [50–100]	< 0.001
Temperature, °C	37.3 [36.5–38.1]	37.5 [37.0–37.8]	37.2 [36.4–38.1]	0.49
Last available biological parameters prior to in-ICU CA				
pH	7.33 [7.22–7.41]	7.40 [7.31–7.43]	7.31 [7.20–7.41]	0.003
PaCO_2_, Torr	51 [40–61]	46 [40–58]	53 [40–62]	0.35
PaO_2_, Torr	73 [60–93]	81 [70–104]	69 [58–90]	0.02
PaO_2_/FiO_2_	119 [67–208]	210 [141–247]	98 [62–175]	< 0.001
Bicarbonate, mmol/l	26 [22–31]	29 [25–31]	25 [22–31]	0.02
Arterial lactate, mmol/l	1.5 [1.0–2.0]	1.1 [0.8–1.7]	1.5 [1.1–2.7]	0.004
Creatinine, μmol/l	117 [59–263]	87 [43–133]	124 [67–275]	0.02
One or more CA confirmed or suspected etiology				
At least one cause identified	132 (90)	30 (100)	102 (88)	0.05
Acute hypoxia	59 (40)	17 (57)	42 (36)	0.06
Endotracheal tube or tracheotomy obstruction	19 (13)	8 (27)	11 (9)	0.18
During intubation procedure	16 (11)	6 (20)	10 (9)	0.48
Unplanned extubation	4 (3)	1 (3)	3 (3)	0.80
Hemoptysis	3 (2)	2 (7)	3 (3)	0.24
Pneumothorax	2 (1)	1 (3)	1 (1)	0.54
Unknown cause	15 (10)	2 (7)	13 (11)	0.09
Refractory septic shock	19 (13)	5 (17)	14 (12)	0.17
Massive pulmonary embolism	24 (16)	1 (3)	23 (20)	0.02
Drug side effect	10 (7)	5 (17)	5 (4)	0.03
Acute metabolic disorder	10 (7)	1 (3)	9 (8)	0.32
Cardiac conductive disorders	7 (5)	3 (10)	4 (3)	0.19
Hemorrhagic shock	2 (1)	0 (0)	2 (2)	0.44
Acute myocarditis	4 (3)	1 (3)	3 (3)	0.91
ECMO dysfunction	5 (3)	0 (0)	5 (4)	0.22
Acute coronary syndrome	3 (2)	1 (3)	2 (2)	0.66
Other etiologies	3 (2)	1 (3)	2 (2)	0.66

Results are expressed as *N* (%) or median [25–75% interquartile range] unless expressed otherwise. Favorable outcome: modified Rankin scale ranging from 0 to 3 at day 90 after CA; unfavorable outcome: modified Rankin scale ranging from 4 to 6 at day 90 after CA; ICU: intensive care unit; CA: cardiac arrest; SOFA: sepsis-related organ failure assessment score; VT/VF: ventricular tachycardia/ventricular fibrillation; ECMO: extracorporeal membrane of oxygenation; FiO2: inspired fraction of oxygen; PaCO_2_: partial pressure of arterial carbon dioxide; PaO_2_: partial pressure of arterial oxygen

^a^Including 12 patients treated with veno-venous ECMO and one patient treated with veno-arterial ECMO

^b^Within one hour before the CA occurrence

Etiologies of in-ICU CA are detailed in Table 2. At least one cause was identified in 132 in-ICU CA patients (90%), hypoxia being the most frequent cause (40%). No significant difference was observed between favorable and unfavorable outcome according to CA etiologies, except for more CA related to massive pulmonary embolism and less CA related to drug side effect in unfavorable outcome compared to favorable outcome (20 vs. 3%, and 4 vs. 17%, respectively).

Before CA occurrence, patients with an unfavorable outcome compared to those with a favorable outcome had more frequently almost 3 organ failures (59 vs. 20%, respectively), including lower PaO_2_/FiO_2_ ratio (98 [62–175] vs. 210 [141–747], respectively), lower mean arterial pressure (73 mmHg [60–87] vs. 86 mmHg [77–94], respectively) and were most frequently treated with vasopressors and renal replacement therapy (53 vs. 40%, and 22 vs. 3%, respectively). Patients with an unfavorable outcome had prolonged low-flow durations and more epinephrine boluses compared to those with a favorable outcome (5 min [2–13] vs. 3 min [1–5], and 2 mg [2–6] vs. 1 mg [1, 2], respectively). SOFA > 9 before CA occurrence was the single parameter constantly and significantly associated with an unfavorable outcome at day 90 after CA in multivariate analysis (Additional file 1: Table S4 in the online supplemental data).

### Organ support and outcomes

Organ support and patients’ outcomes are detailed in Table 3. Among the 146 patients with CPR attempt, 102 (70%) patients died in the ICU, including 48 (33%) patients dying within the first day after CA occurrence, 29 (20%) dying without sustained ROSC and 21 (14%) patients dying from WLST. At day 90 after CA, 40 patients (27%) survived, and 30 patients (21%) had a favorable outcome. Among patients with CPR attempt, favorable outcome rates were, respectively, 63%, 18%, 18% and 0% in patients < 45 years, between 45–64 years, between 65–79 years, and > 79 years (Additional file 1 Figure S1 in the online supplemental data).

**Table 3:** Organ support and clinical outcomes in the cohort according to favorable or unfavorable outcome at day 90 after in-ICU CA

Variable	All patients (*N* = 146)	Favorable (*N* = 30)	Unfavorable (*N* = 116)	*p* value
Organ support				
Duration of mechanical ventilation, days	15 [7–36]	33 [19–45]	14 [5–28]	< 0.001
Vasopressors	109 (82)	25 (83)	84 (72)	0.06
Duration of vasopressor treatment, days	6 [3–11]	7 [4–16]	6 [2–11]	0.09
Neuromuscular blocking agent	131 (90)	26 (87)	105 (91)	0.54
Prone positioning	111 (76)	24 (80)	87 (75)	0.57
Renal replacement therapy	66 (45)	12 (40)	54 (47)	0.52
Duration of renal replacement therapy, days	5 [2–11]	6 [3–21]	5 [2–10]	0.29
ECMO	20 (14)	4 (13)	16 (14)	0.95
Targeted temperature management	7 (5)	3 (10)	4 (3)	0.13
Outcome at ICU discharge				
Death	102 (70)	–	102 (88)	–
New in-ICU CA occurrence	31 (21)	–	31 (27)	–
Unsuccessful CPR attempt	29 (20)	–	29 (25)	–
From WLST	21 (14)^a^	–	21 (18)	–
From multi-organ failure	18 (12)	–	18 (16)	–
From refractory ARDS	3 (2)	–	3 (3)	–
Duration of ICU hospitalization, days	20 [8–42]	45 [28–71]	16 [7–33]	< 0.001
Outcome at day 90 after in-ICU CA				
Died	106 (73)	–	106 (91)	-
Remained hospitalized	2 (1)	0 (0)	2 (2)	0.47
Duration of hospitalization, days	26 [10–54]	59 [46–94]	17 [8–38]	< 0.001
Modified Rankin scale				
0	6 (4)	6 (20)	–	–
1	9 (6)	9 (30)	–	–
2	10 (7)	10 (33)	–	–
3	5 (3)	5 (16)	–	–
4	6 (4)	–	6 (7)	–
5	4 (3)	–	4 (3)	–

Results are expressed as N (%) or median [25–75% interquartile range] unless expressed otherwise. Favorable: modified Rankin scale ranging from 0 to 3 ay day 90 after CA; Unfavorable: modified Rankin scale ranging from 4 to 6 at day 90 after CA; ICU: intensive care unit; CA: cardiac arrest; ECMO: extracorporeal membrane of oxygenation; CPR: cardiopulmonary resuscitation; WLST: withdrawal of life-sustaining therapy; ARDS: acute respiratory distress syndrome

^a^Including 4 WLST for neurological impairment and 17 WLST for other reasons

**Fig. 2:**
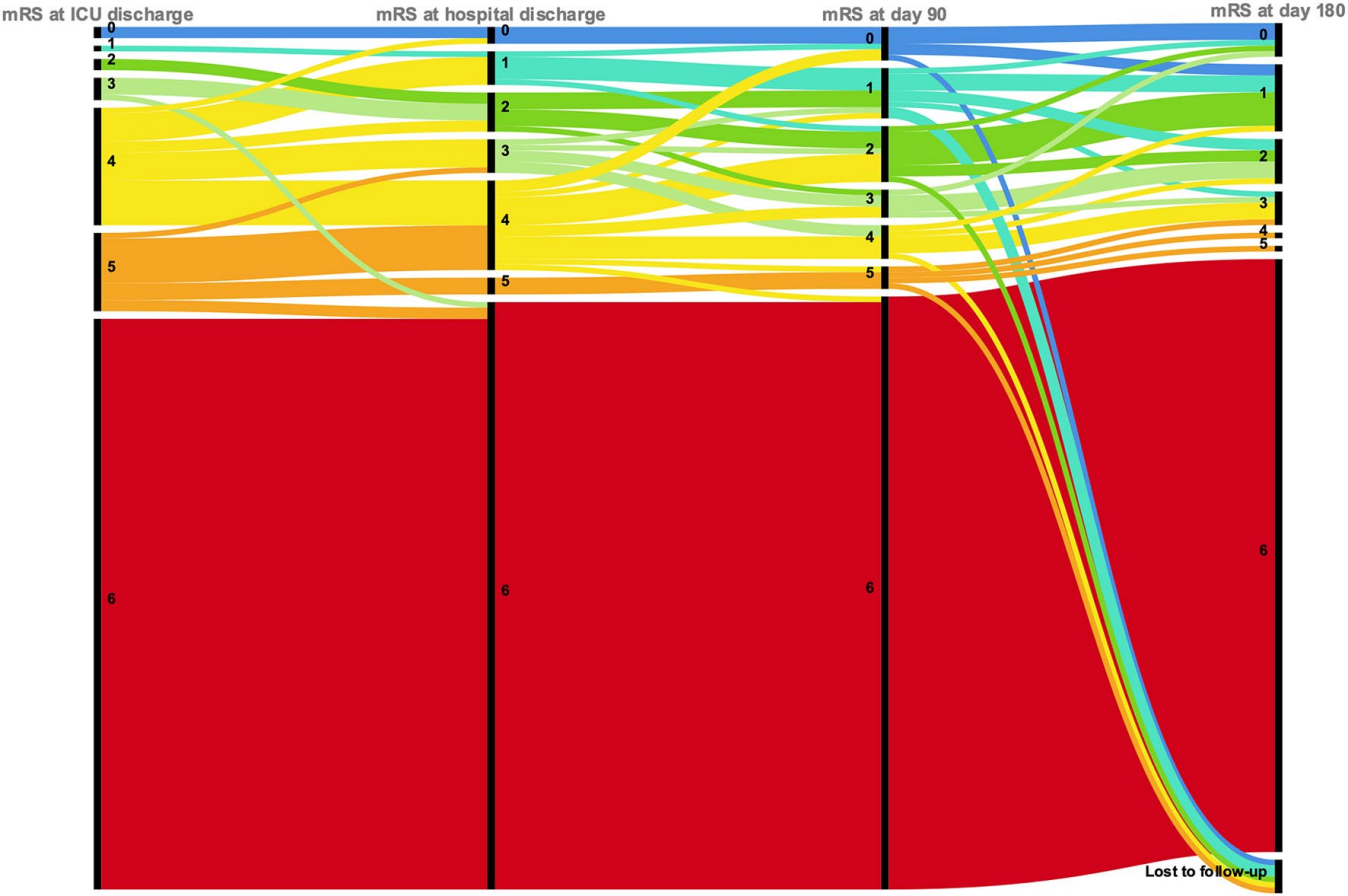
Alluvial diagram of patients’ outcome at ICU and hospital discharge, at day 90 and 180 after CA. *ICU* intensive care unit, *CA* cardiac arrest, *mRS* modified Rankin scale

Six patients out of 40 were lost to follow-up at day 180 (4 and 2 patients in the favorable and unfavorable outcome group, respectively). Among the 8 remaining patients who survived at hospital discharge with unfavorable outcome at day 90, 6 patients improved to a good functional status at day 180. All of 26 remaining patients with good functional status at day 90 were still experiencing favorable outcome at day 180 (Fig. 2).

## Discussion

### Main findings

In this multicenter cohort screening 2425 COVID-19 French adult patients, 6% experienced in-ICU CA of whom 80% had sustained ROSC. Initial rhythm was mostly non-shockable, and hypoxia was the most frequent CA etiology. More than 20% patients with CPR survived with good functional status at day 90 or thereafter. SOFA > 9 before CA occurrence was the single factor significantly and constantly associated with unfavorable outcome.

### Current literature: in-ICU CA incidence and outcome

In-ICU CA incidence varied before the COVID-19 outbreak from 0.5 to 5% according to previous reports [1–4]. In the recent French multicenter “ACIR” study including 31.399 ICU patients admitted for shock, respiratory failure, out-of-hospital or in-hospital resuscitated CA outside COVID-19, 677 patients (2%) experienced in-ICU CA with CPR attempt [3], compared to 6% of COVID-19 patients in our study. In-ICU CA seems common in hospitalized COVID-19 patients, and possibly be more frequent than in other pathologies. Indeed, the incidence of overall in-ICU CA and those with CPR attempt was 14, and 8%, respectively, in the only other published multicenter in-ICU CA cohort of patients admitted for COVID-19 in USA [16]. Moreover, a recent review of the incidence of IHCA in patient with COVID-19 reported between 8 and 11% among ICU patients [22].

Regarding outcome of in-ICU CA patients, our results are similar to those published before the COVID-19 outbreak. In the previously cited “ACIR” cohort, 118 patients (17%) were alive at 6 months with good functional status [3], which is concordant with our results. However, all the recent small or monocentric reports regarding IHCA in COVID-19 patients outcome have described very low survival rates at discharge, near or equal to 0% even in patients with sustained ROSC [5–7]. This major difference with our results regarding the global outcome does not seem related to CA etiologies: as observed in our study, non-shockable rhythm and hypoxia were also described as the most frequent initial rhythm and etiology responsible for in-ICU CA [5–8, 16, 17]. Conversely, this difference in outcome could mainly be explained by our inclusion criteria and selection of patients: only in-ICU CA patients with CPR attempt have been included here in our study. By contrast, most of previous studies have included a limited number of in-ICU CA (with or without CPR attempt) or only IHCA in unmonitored wards which are known to be associated with poorer outcome [22, 23].

In the largest multicenter cohort of 701 in-ICU CA patients admitted for COVID-19 in the USA, 400 patients (57%) received CPR (the 301 remaining had a “do not resuscitate” status at the time of CA) of whom 135 (34%) had sustained ROSC and only 28 (7%) survived at hospital discharge with good neurological status [16]. Our study reports a relative higher proportion of patients with CPR attempt (78%), sustained ROSC (80%), and higher survival rate with good functional status (21%). Several differences between our study and the study published by Hayek et al. can be noted, despite similar median age and SOFA at ICU admission. First, Hayek and co-workers have only included in-ICU CA occurring during the early phase of COVID-19 history (i.e., within 14 days from ICU admission) with a mean ICU length of stay of 6 days. Conversely in our cohort, the median ICU length of stay was 20 days, and CA occurred later than 14 days after ICU admission in patients with good outcome. Moreover, patients’ therapeutic management performed in the Hayek’s study was different compared to our study: more patients received almost two vasopressors (52 vs. 4%), but less patients were mechanically ventilated (72 vs. 90%), and less patients were treated with ECMO at CA occurrence (0.5 vs. 9%). This suggests that CA occurred possibly at a later stage of COVID-19 in our population, and that the delay between ICU admission and CA occurrence may have influenced outcome in such patients. Finally, we reported that almost 18% of patients with unfavorable outcome died in ICU from WLST. Although WLST decisions during the post-CA period could also explain the discrepancies between Hayek’s study and ours regarding patients’ outcome, this data was not described by Hayek and co-workers.

Patients’ age is another important parameter that could partly explain our higher favorable outcome rate. Indeed, in the study published by Hayek and co-workers where age ≥ 80 years was independently associated with an increased risk of death, patients with CPR attempt and favorable outcome were younger compared to those with CPR attempt and unfavorable outcome [16]. Despite similar median age between our cohort and the Hayek’s study, our univariate analysis did not find any difference regarding patients’ age and outcome. This difference could be explained by the low proportion of patients ≥ 80 years requiring CPR attempt in our cohort compared to patients described in the Hayek’s study (1 vs. 8%, respectively). In another multicenter cohort of 260 IHCA in COVID-19 patients, Mitchell et al. have also found that older patients were at higher risk of 30-day mortality [17]. However, several differences with our study can also be described: authors have included older patients than those of our cohort (median age 69 vs. 63 years, respectively), and a large proportion of patients (36%) experienced CA in unmonitored wards, whereas all our CA patients were monitored.

### Practical implications

Finally in our study, among the 10 patients of our cohort with mRS ≥ 4 at day 90 after CA, six patients later improved to a good functional status at 6 months. Considering these latter six patients, the overall favorable outcome rate should finally reach 25% of patients with CPR attempt and 31% of patients with sustained ROSC. These results as other previous cited works [16, 17] strongly suggest to not systematically apply a “do-not-resuscitate” order among ICU patients with severe COVID-19, and to promote specific and safe CPR protocols for healthcare givers. More studies are warranted to clearly identify factors to guide physicians in their decisions to initiate or not CPR in such patients.

### Limitations

Several limitations in our study also must be acknowledged. First, we did not assess the ICU overflow of the participating centers during the study period. Indeed, Hayek et al. also identified admission to hospital with a smaller number of ICU beds as a risk factor for higher in-ICU CA incidence and mortality [16]. This result has probably highlighted the impact of the ICU overflow on COVID-19 patient’s outcome. However, there were no differences in our study between ICUs according to their type and number of beds. Second, all data were collected during the first months of the COVID-19 outbreak in France and might not reflect current practices regarding this pathology, since non-invasive oxygenation strategies or corticosteroids use have been modified during time. Third, a very small number of patients with sustained ROSC were treated using targeted temperature management in our cohort although recent data suggest that it may be effective especially in CA with non-shockable rhythm [24]. Fourth, despite that most CA in our cohort were related to COVID-19 consequences (acute hypoxia, pulmonary embolism, etc.), our results could not clearly differentiate deaths related to COVID-19 severity versus those related to post-CA syndrome and help to better understand these different and often intricate mechanisms of mortality. Moreover, our study was not designed to predict survival with poor outcome, which is related to significant workload and high treatment costs [25]. Fifth, results of our multivariate analysis must be carefully interpreted. Indeed, our limited sample size cohort only allowed us to implement a limited number of significant and clinically relevant parameters in our multivariate analysis, as generally recommended [26]. As in our study, Leloup et al. also identified before the COVID-19 outbreak, SOFA before in-ICU CA occurrence as a risk factor associated with an unfavorable outcome [3]. But regardless of high SOFA or multi-organ failure diagnosis, our study confirms that the presence of simple abnormal parameters prior to in-ICU CA, such as PaO_2_ or FiO_2_ setting, seems also relevant to predict COVID-19 patients’ outcome, as previously described [27]. Sixth, we did not assess patients’ functional capacity before CA. However, previous studies regarding IHCA patients without COVID-19, or COVID-19 patients regardless of CA occurrence, have already suggested a statistical association between such parameters (like age, comorbidities and functional status prior to ICU admission) and patients’ outcome [28, 29]. Finally, we have exclusively included in-ICU CA, and our results cannot be extrapolated in IHCA occurring in unmonitored wards.

## Conclusion

In-ICU CA incidence remains high among adults with COVID-19. However, 21% of patients with CPR attempt were alive at 3 months follow-up with good functional status. This suggests to not systematically apply a “do-not-resuscitate” order among ICU patients with severe COVID-19.

## Supplementary Information

**Additional file 1**:** Table S1**. Main characteristics of the 35 participating intensive care units (ICU).** Table S2**. Other general characteristics at ICU admission of overall patients and according to outcome at day 90 after in-ICU CA.** Table S3**. Characteristics at in-ICU CA occurrence of the overall cohort and according to outcome at day 90 after CA.** Table S4**. Multivariate analysis of factors associated with unfavorable outcome among the 146 patients with CPR.** Fig. S1**. Proportion of CPR underwent among ICU patients with CA occurrence and proportion of favorable outcome among patients who underwent CPR, stratified by age.** Fig. S2**. Percentage of patients with unfavorable outcome according to SOFA score before in-ICU CA occurrence.

## Data Availability

Research data and other material will be made available to the scientific community, immediately on publication, with as few restrictions as possible. All requests should be submitted to the corresponding author who will review with the other investigators for consideration. A data use agreement will be required before the release of participant data and institutional review board approval as appropriate.

## Abbreviations

CA: Cardiac arrest
CI: Confidence interval
COVID-19: Coronavirus disease 2019
CPR: Cardiopulmonary resuscitation
FiO_2_: Fraction of inspired oxygen
ICU: Intensive care unit
IHCA: In-hospital cardiac arrest
mRS: Modified Rankin scale
MV: Mechanical ventilation
OR: Odds ratio
PaO_2_: Arterial partial pressure of oxygen
ROSC: Return of spontaneous circulation
SAPS-2: Simplified Acute Physiology Score 2
SOFA: Sepsis-related Organ Failure Assessment Score
WLST: Withdrawal of life-sustaining therapy
