# Supplementary material for: Incidence, clinical characteristics, and outcome after unexpected cardiac arrest among critically ill adults with COVID-19: insight from the multicenter prospective ACICOVID-19 registry
Source: Ann Intensive Care. 2021 Nov 13;11:155. doi: 10.1186/s13613-021-00945-y (PMC8590126; doi:10.1186/s13613-021-00945-y)
Supplement: Supplementary file 1 — Additional file 1: Table S1. Main characteristics of the 35 participating intensive care units (ICU). Table S2. Other general characteristics at ICU admission of overall patients and according to outcome at day 90 after in-ICU CA. Table S3. Characteristics at in-ICU CA occurrence of the overall cohort and according to outcome at day 90 after CA. Table S4. Multivariate analysis of factors associated with unfavorable outcome among the 146 patients with CPR. Fig. S1. Proportion of CPR underwent among ICU patients with CA occurrence and proportion of favorable outcome among patients who underwent CPR, stratified by age. Fig. S2. Percentage of patients with unfavorable outcome according to SOFA score before in-ICU CA occurrence. [file 13613_2021_945_MOESM1_ESM.docx]

**Incidence, clinical characteristics, and outcome after unexpected cardiac arrest among critically ill adults with COVID-19: insight from the multicenter prospective ACICOVID-19 registry.**

Jonathan Chelly, Gaetan Plantefève, Toufik Kamel, Cédric Bruel, Saad Nseir, Christopher Lai, Giulia Cirillo, Elena Skripkina, Sébastien Ehrminger, Fernando-Daniel Berdaguer-Ferrari, Julien Le Marec, Marine Paul, Aurélie Autret, Nicolas Deye, on behalf the ACICOVID-19 study group.

**
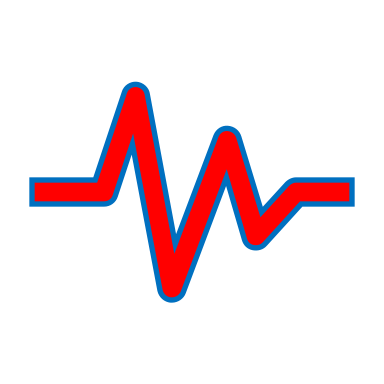

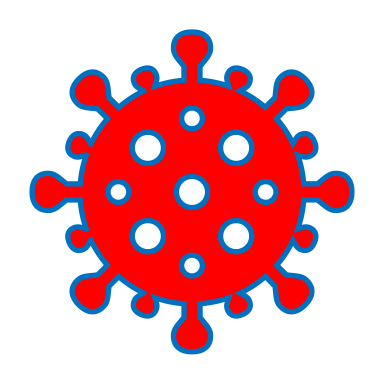
**

**ACICOVID-19 study group**

**ONLINE SUPPLEMENTAL DATA**

**TABLE AND FIGURE LEGEND**

**Table S1.** Main characteristics of the 35 participating intensive care units (ICU).

**Table S2.** Other general characteristics at ICU admission of overall patients and according to outcome at day 90 after in-ICU CA.

**Table S3.** Characteristics at in-ICU CA occurrence of the overall cohort and according to outcome at day 90 after CA.

**Table S4.** Multivariate analysis of factors associated with unfavorable outcome among the 146 patients with CPR.

**Figure S1.** Proportion of CPR underwent among ICU patients with CA occurrence and proportion of favorable outcome among patients who underwent CPR, stratified by age.

**Figure S2.** Percentage of patients with unfavorable outcome according to SOFA score before in-ICU CA occurrence.

**Table S1. Main characteristics of the 35 participating ICUs**.

| **Characteristics** | **Participating ICUs** |
| --- | --- |
| **Hospital type – n (%)** |  |
| University hospital | 15 (43) |
| Public regional | 16 (45) |
| Semi-private | 2 (6) |
| Private | 2 (6) |
| **ICU type – n (%)** |  |
| Medical | 12 (34) |
| Medical-surgical | 21 (60) |
| Neuro-surgical | 1 (3) |
| Cardiac and thoracic | 1 (3) |
| **Number of ICU beds** |  |
| Median [interquartile range] | 27 [20 – 39] |
| < 12 beds – n (%) | 1 (3) |
| 12 – 18 beds – n (%) | 6 (17) |
| 19 – 24 beds – n (%) | 8 (23) |
| 25 – 32 beds – n (%) | 10 (28) |
| 33 – 50 beds – n (%) | 7 (20) |
| > 50 beds – n (%) | 3 (9) |
| Results are expressed as N (%) or median [25-75% interquartile range] unless expressed otherwise. ICU: intensive care unit | |

**Table S2. Other general characteristics at ICU admission of overall patients and according to outcome at day 90 after in-ICU CA.**

| ***Characteristic*** | ***All patients***  ***(N = 146)*** | ***Favorable***  ***(N = 30)*** | | ***Unfavorable***  ***(N = 116)*** | ***p***  ***value*** |
| --- | --- | --- | --- | --- | --- |
| **Hospital and ICU type** |  |  |  | |  |
| University hospital | 77 (53) | 16 (53) | 61 (53) | | 0.94 |
| ICU type |  |  |  | | 0.24 |
| Medico-surgical | 71 (49) | 15 (50) | 56 (48) | |  |
| Medical | 65 (44) | 15 (50) | 50 (43) | |  |
| Surgical | 10 (7) | 0 (0) | 10 (9) | |  |
| **Covid-19 symptoms at hospital admission** |  |  |  | |  |
| Interval from first symptoms to hospital admission – days | 7 [4 – 10] | 7 [5 – 10] | 6 [4 – 10] | | 0.46 |
| Interval from first symptoms to ICU admission – days | 9 [6 – 12] | 9 [7 – 12] | 8 [5 – 12] | | 0.42 |
| Shortness of breath | 134 (92) | 26 (87) | 108 (93) | | 0.25 |
| Fever | 120 (82) | 29 (97) | 91 (78) | | 0.02 |
| Digestive symptoms | 36 (25) | 7 (23) | 29 (25) | | 0.85 |
| Gustatory or olfactory dysfunction | 28 (19) | 6 (20) | 22 (19) | | 0.90 |
| Chest pain | 6 (4) | 0 (0) | 6 (5) | | 0.20 |
| **Covid-19 antiviral treatment prior to in-ICU CA occurrence** | 75 (51) | 21 (70) | 54 (47) | | 0.02 |
| Still in progress at the time of in-ICU CA | 19 (13) | 7 (23) | 12 (10) | | 0.32 |
| Location of initiation |  |  |  | |  |
| ICU | 46 (32) | 14 (47) | 32 (28) | | 0.35 |
| Ward | 29 (20) | 7 (23) | 22 (19) | | 0.59 |
| Continuation after ICU admission | 18 (12) | 6 (20) | 12 (10) | | 0.56 |
| Treatment used |  |  |  | |  |
| Hydroxychloroquine | 40 (27) | 13 (43) | 27 (23) | | 0.35 |
| Azithromycin | 25 (17) | 6 (20) | 19 (16) | | 0.59 |
| Lopinavir/Ritonavir | 20 (14) | 5 (17) | 15 (13) | | 0.73 |
| Ascorbic acid | 9 (6) | 5 (17) | 4 (3) | | 0.24 |
| Oseltamivir | 6 (4) | 2 (7) | 4 (3) | | 0.76 |
| Tocilizumab | 5 (3) | 2 (7) | 3 (3) | | 0.54 |
| Anakinra | 3 (2) | 0 (0) | 3 (3) | | 0.27 |
| Results are expressed as N (%) or median [25-75% interquartile range] unless expressed otherwise. Favorable: modified Rankin scale ranging from 0 to 3 at day 90 after CA; Unfavorable: modified Rankin scale ranging from 4 to 6 at day 90 after CA; ICU: intensive care unit; CA: cardiac arrest. | | | | | |

**Table S3. Other characteristics at in-ICU CA occurrence according to outcome at day 90.**

| ***Characteristic*** | ***All patients***  ***(N = 146)*** | ***Favorable***  ***(N = 30)*** | ***Unfavorable***  ***(N = 116)*** | ***p***  ***value*** |
| --- | --- | --- | --- | --- |
| **Ongoing treatments at the time of CA** |  |  |  |  |
| Mechanical ventilation with tracheotomy cannula | 5 (3) | 2 (7) | 3 (3) | 0.29 |
| PEEP level under mechanical ventilation | 10 [7 – 12] | 10 [7 – 12] | 10 [7 – 13] | 0.16 |
| High flow nasal canula oxygen therapy | 4 (3) | 1 (3) | 3 (3) | 0.82 |
| O_2_ therapy with bag-valve-mask (10 to 15 lpm) | 3 (2) | 0 (0) | 3 (3) | 0.37 |
| O_2_ therapy with non-rebreathable face mask (6 to 9 lpm) | 3 (2) | 0 (0) | 3 (3) | 0.37 |
| O_2_ therapy with nasal canula (1 to 5 lpm) | 2 (1) | 1 (3) | 1 (1) | 0.30 |
| Non-invasive ventilation | 2 (1) | 1 (3) | 1 (1) | 0.30 |
| Norepinephrine continuous infusion | 64 (44) | 11 (37) | 53 (46) | 0.37 |
| Epinephrine continuous infusion | 10 (7) | 0 (0) | 10 (9) | 0.22 |
| ≥ 2 vasopressors infusions simultaneously | 6 (4) | 0 (0) | 6 (5) | 0.35 |
| Dobutamine continuous infusion | 1 (1) | 0 (0) | 1 (1) | 0.99 |
| **Other suspected or confirmed CA etiology** |  |  |  |  |
| Acute mesenteric ischemia | 1 (1) | 0 (0) | 1 (1) | 0.24 |
| Acute cardiac tamponade | 1 (1) | 1 (3) | 0 (0) | 0.11 |
| Acute stroke | 1 (1) | 0 (0) | 1 (1) | 0.24 |
| Results are expressed as N (%) or median [25-75% interquartile range] unless expressed otherwise. Favorable: modified Rankin scale ranging from 0 to 3 at day 90 after CA; Unfavorable: modified Rankin scale ranging from 4 to 6 at day 90 after CA; ICU: intensive care unit; CA: cardiac arrest; PEEP: positive end-expiratory pressure; O_2_: oxygen; lpm: litter per minute. | | | | |

**Table S4. Multivariate analysis of factors associated with unfavorable outcome among the 146 patients with CPR.**

| **Variable** | **Unadjusted**  **OR** | **[95% CI]** | ***p***  ***value*** | **Adjusted**  **OR** | **[95% CI]** | ***p***  ***value*** |
| --- | --- | --- | --- | --- | --- | --- |
| **Step 1** |  |  |  |  |  |  |
| SOFA > 9 before CA occurrence | 7.37 | [2.87 – 18.87] | < 0.001 | 10.13 | [1.82 – 56.46] | 0.008 |
| Arterial lactate level – mmol/l ^a^ | 2.17 | [1.09 – 4.35] | 0.004 | 4.10 | [0.84 – 20.08] | 0.08 |
| Total epinephrine bolus during CPR – mg | 1.43 | [1.06 – 1.93] | < 0.001 | 1.49 | [0.89 – 2.47] | 0.13 |
| PaO_2_/FiO_2_ ratio – per 10 mmHg decrease ^a^ | 0.94 | [0.91 – 0.98] | 0.001 | 0.95 | [0.89 – 1.02] | 0.16 |
| Mean arterial pressure – mmHg ^a^ | 0.97 | [0.99 – 0.99] | < 0.001 | 0.97 | [0.93 – 1.02] | 0.23 |
| pH ^b^ | 0.00 | [0.00 – 0.18] | 0.003 | 133.48 | [0.03 – >100] | 0.25 |
| Low flow – min | 1.01 | [1.01 - 1.18] | 0.004 | 1.00 | [0.90 – 1.11] | 0.99 |
| **Step 2** |  |  |  |  |  |  |
| SOFA > 9 before CA occurrence | - | - | - | 12.21 | [2.18 – 68.26] | 0.004 |
| Total epinephrine bolus during CPR – mg | - | - | - | 1.58 | [1.04 – 2.39] | 0.20 |
| Arterial lactate level – mmol/l ^a^ | - | - | - | 4.54 | [0.99 – 20.81] | 0.05 |
| PaO_2_/FiO_2_ ratio – per 10 mmHg decrease ^a^ | - | - | - | 0.95 | [0.88 – 1.01] | 0.12 |
| Mean arterial pressure – mmHg ^a^ | - | - | - | 0.97 | [0.93 – 1.02] | 0.25 |
| pH | - | - | - | 137.65 | [0.03 – >100] | 0.26 |
| **Step 3** |  |  |  |  |  |  |
| SOFA > 9 before CA occurrence | - | - | - | 8.10 | [1.80 – 36.54] | 0.006 |
| Total epinephrine bolus during CPR – mg | - | - | - | 1.47 | [1.02 – 2.13] | 0.04 |
| Arterial lactate level – mmol/l ^a^ | - | - | - | 4.03 | [0.96 – 16.97] | 0.06 |
| PaO_2_/FiO_2_ ratio per 10 mmHg decrease ^a^ | - | - | - | 0.95 | [0.89 – 1.02] | 0.14 |
| Mean arterial pressure – mmHg ^a^ | - | - | - | 0.99 | [0.95 – 1.02] | 0.48 |
| **Step 4** |  |  |  |  |  |  |
| SOFA > 9 before CA occurrence | - | - | - | 8.27 | [1.95 – 35.08] | 0.004 |
| Total epinephrine bolus during CPR – mg | - | - | - | 1.58 | [1.05 – 2.38] | 0.03 |
| Arterial lactate level – mmol/l ^a^ | - | - | - | 3.11 | [0.85 – 11.33] | 0.09 |
| PaO_2_/FiO_2_ ratio – per 10 mmHg decrease ^a^ | - | - | - | 0.97 | [0.91 – 1.03] | 0.26 |
| **Step 5** |  |  |  |  |  |  |
| SOFA > 9 before CA occurrence | - | - | - | 10.57 | [2.70 – 41.48] | 0.001 |
| Total epinephrine bolus during CPR – mg | - | - | - | 1.56 | [1.06 – 2.31] | 0.03 |
| Arterial lactate level – mmol/l ^a^ | - | - | - | 2.63 | [0.79 – 8.75] | 0.12 |
| Unfavorable outcome: modified Rankin scale ranging from 4 to 6 at day 90 after CA; CA: cardiac arrest; SOFA score: sepsis organ failure assessment score; CPR: cardiopulmonary resuscitation; OR: Odds ratio; CI: confidence interval.  ^a^ Last known variable before cardiac arrest occurrence | | | | | | |

**Figure S1. Proportion of CPR underwent among ICU patients with CA occurrence and proportion of favorable outcome among patients who underwent CPR, stratified by age.** (Ratio between CPR attempt and CA occurrence, and between favorable outcome and CPR attempts given for each age category; ICU: intensive care unit; CA: cardiac arrest; CPR: cardiopulmonary resuscitation; Favorable outcome: defined as modified Rankin scale ranging from 0 to 3 at day 90 after CA. Comparison according to each age category was performed using Pearson Chi-squared test; * p < 0.001; ** p = 0.02)

**Figure S2. Percentage of patients with unfavorable outcome according to SOFA score before in-ICU CA occurrence.**

(SOFA score: sepsis organ failure assessment score; ICU: intensive care unit; CA: cardiac arrest; Unfavorable outcome: defined as modified Rankin scale between 4 and 6 at day 90 after CA).
